# Telesurgery and the Future of Urologic Surgery

**DOI:** 10.1590/S1677-5538.IBJU.2025.06.01

**Published:** 2025-10-06

**Authors:** Luciano A. Favorito

**Affiliations:** 1 Universidade do Estado do Rio de Janeiro Unidade de Pesquisa Urogenital Rio de Janeiro RJ Brasil Unidade de Pesquisa Urogenital - Universidade do Estado do Rio de Janeiro - Uerj, Rio de Janeiro, RJ, Brasil;; 2 Hospital Federal da Lagoa Serviço de Urologia Rio de Janeiro RJ Brasil Serviço de Urologia, Hospital Federal da Lagoa, Rio de Janeiro, RJ, Brasil

The November–December 2025 issue of the International Brazilian Journal of Urology presents original contributions with numerous engaging papers across various fields, including urinary incontinence, bladder cancer, prostate cancer, robotic surgery, telesurgery in urology, infection, penile cancer, infertility, transitional urology, epispadias, and basic research. The papers come from several countries such as Brazil, Kuwait, Serbia, Italy, and China. As usual, the Editor's Comment highlights selected articles, and in this issue, the Editor-in-Chief wishes to particularly emphasize the paper on telesurgery.

Dr. Aldousari and colleagues from Kuwait present, on page e20250331 (1), an interesting study on telesurgery, which is featured on the cover of this edition. The objective of their work was to demonstrate the feasibility and reproducibility of ultra–long-distance, Asia-to–Middle East human telesurgery using low-latency connectivity, performed on multiple patients and across different procedures. Over a six-month period, the authors performed five human telesurgeries using a multiport robotic platform connected via fiber-optic and 5G networks. The remote surgeon (SA) was located in Shanghai, China, while the patients were in Kuwait City, Kuwait—approximately 7,000 kilometers apart. They concluded that multiple human telesurgical procedures can be feasibly and reproducibly performed through successful collaboration between two countries using a robotic system with telecommunication capabilities, achieving favorable outcomes without complications. However, they also emphasize the need to establish robust international guidelines to facilitate the global implementation of telesurgery.

In recent years, we have witnessed remarkable advancements in robotic surgery and other emerging technologies in urology (2–6). Among them, telesurgery stands out as the most striking innovation. This technology has enabled a previously unimaginable achievement in surgery: the ability for a surgeon to perform a procedure from a completely different location—another city, country, or even continent. This pioneering progress is largely due to the dedicated work of Dr. Patel, Dr. Moschovas, and their team (7). The demonstrated feasibility of long-distance telesurgery not only marks a milestone in urologic surgery but also underscores its potential to improve surgical outcomes, facilitate advanced training, and provide remote assistance for complex cases. Telesurgery could play a pivotal role in expanding access to specialized care and enhancing robotic surgical training worldwide.

The Editor-in-chief expects everyone to enjoy reading the original contributions with a lot of interesting papers in this number.
